# *Schistosoma mansoni* phosphoglycerate mutase: a glycolytic ectoenzyme with thrombolytic potential[Fn FN1]

**DOI:** 10.1051/parasite/2022042

**Published:** 2022-09-09

**Authors:** David B. Pirovich, Akram A. Da’dara, Patrick J. Skelly

**Affiliations:** 1 Molecular Helminthology Laboratory, Department of Infectious Disease and Global Health, Cummings School of Veterinary Medicine, Tufts University 200 Westboro Road North Grafton MA 01536 USA

**Keywords:** Schistosomiasis, Tegument, PGM, Moonlighting function, Thrombolysis, Glycolytic enzyme

## Abstract

Schistosomiasis is a debilitating parasitic disease caused by intravascular flatworms called schistosomes (blood flukes) that affects >200 million people worldwide. Proteomic analysis has revealed the surprising presence of classical glycolytic enzymes – typically cytosolic proteins – located on the extracellular surface of the parasite tegument (skin). Immunolocalization experiments show that phosphoglycerate mutase (PGM) is widely expressed in parasite tissues and is highly expressed in the tegument. We demonstrate that live *Schistosoma mansoni* parasites express enzymatically active PGM on their tegumental surface. Suppression of PGM using RNA interference (RNAi) diminishes *S. mansoni* PGM (SmPGM) gene expression, protein levels, and surface enzyme activity. Sequence comparisons place SmPGM in the cofactor (2,3-bisphosphoglycerate)-dependent PGM (dPGM) family. We have produced recombinant SmPGM (rSmPGM) in an enzymatically active form in *Escherichia coli*. The Michaelis-Menten constant (*K*_m_) of rSmPGM for its glycolytic substrate (3-phosphoglycerate) is 0.85 mM ± 0.02. rSmPGM activity is inhibited by the dPGM-specific inhibitor vanadate. Here, we show that rSmPGM not only binds to plasminogen but also promotes its conversion to an active form (plasmin) *in vitro*. This supports the hypothesis that host-interactive tegumental proteins (such as SmPGM), by enhancing plasmin formation, may help degrade blood clots around the worms in the vascular microenvironment and thus promote parasite survival *in vivo*.

## Abstract (fr)

La schistosomiase est une maladie parasitaire débilitante causée par des vers plats intravasculaires appelés schistosomes qui affecte plus de 200 millions de personnes dans le monde. L’analyse protéomique a révélé la présence surprenante d’enzymes glycolytiques classiques – typiquement des protéines cytosoliques – situées sur la surface extracellulaire du tégument du parasite. Des expériences d’immunolocalisation montrent que la phosphoglycérate mutase (PGM) est largement exprimée dans les tissus parasitaires et fortement exprimée dans le tégument. Nous démontrons que les parasites *Schistosoma mansoni* vivants expriment une PGM enzymatiquement active sur leur surface tégumentaire. La suppression de la PGM à l’aide de l’interférence ARN (ARNi) diminue l’expression du gène PGM de *S. mansoni* (SmPGM), les niveaux de protéines et l’activité enzymatique de surface. Les comparaisons de séquences placent la SmPGM dans la famille des PGM dépendantes du cofacteur (2,3-bisphosphoglycérate) (dPGM). Nous avons produit de la SmPGM recombinante (rSmPGM) sous une forme enzymatiquement active dans *Escherichia coli*. La constante de Michaelis-Menten (Km) de rSmPGM pour son substrat glycolytique (3-phosphoglycérate) est de 0,85 mM ± 0,02. L’activité de la rSmPGM est inhibée par le vanadate, un inhibiteur spécifique des dPGM. Ici, nous montrons que rSmPGM non seulement se lie au plasminogène mais favorise également sa conversion en une forme active (plasmine) *in vitro*. Cela soutient l’hypothèse selon laquelle les protéines tégumentaires interactives avec l’hôte (telles que SmPGM), en améliorant la formation de plasmine, peuvent aider à dégrader les caillots sanguins autour des vers dans le microenvironnement vasculaire et ainsi favoriser la survie du parasite *in vivo*.

## Introduction

Schistosomiasis is a neglected tropical disease caused by parasitic blood flukes (flatworms) in the genus *Schistosoma* that currently infect over 200 million people in over 70 countries worldwide [26, 31]. Among parasitic diseases, schistosomiasis is considered second only to malaria in its global morbidity and socioeconomic impact [47]: current estimates of the disability adjusted life-years (DALYs) incurred by schistosomiasis are between 1.7 and 4.5 million [29]. Infection has an estimated mortality rate of ~290,000 deaths per year [31]. The three major schistosome species which infect humans are *Schistosoma mansoni* (endemic to Africa, South America, and the Arabian Peninsula), *Schistosoma japonicum* (endemic to China, Indonesia, and the Philippines), and *Schistosoma haematobium* (endemic to Africa and the Arabian Peninsula) [18, 29]. Infection begins when free-swimming larvae (cercariae) penetrate the skin of mammalian hosts where they transform into juvenile stages called schistosomula [9]. These enter the vasculature and travel to the liver, where the parasites mature into adults [32]. Adult male and female worms pair off and migrate from the portal vein to their egg-laying sites, either the mesenteric veins of the intestine (*S. mansoni* and *S. japonicum*) or the venous plexus of the bladder (*S. haematobium*) [32].

Our laboratory focuses on characterizing the molecular makeup of the worm’s host interactive tegument (skin). In addition to the detection of transporter proteins and immunoregulatory enzymes in the schistosome tegument, proteomic analysis also indicates the somewhat surprising presence at the host-parasite interface of parasite glycolytic enzymes [36]. This is surprising since glycolysis is a highly conserved sequence of ten enzyme-catalyzed reactions that takes place in the cytosol of most organisms. Glycolysis drives the conversion of glucose to pyruvate and results in the generation of the high-energy molecule adenosine triosephosphate (ATP) and NADH – a reduced form of nicotinamide adenine dinucleotide (NAD) [8]. The notion that some glycolytic enzymes are also located on the external surface of schistosomes requires careful examination, especially since any parasite damage in culture could lead to the release or leakage of cytosolic proteins (like glycolytic enzymes), resulting in the erroneous conclusion that these enzymes are also present within the tegument at the host-interactive surface [50].

In previous work, we have detected minimal leakage or secretion of two previously characterized *S. mansoni* glycolytic enzymes – enolase (SmEno) [13] and glyceraldehyde-3-phosphate dehydrogenase (SmGAPDH) from cultured parasites [35]. Moreover, we find substantial SmEno and SmGAPDH enzyme activities associated with living worms in culture, providing strong evidence that these enzymes at least are not released to any substantial degree under our *in vitro* culture conditions but are bound to the exterior of intravascular stage schistosomes [13, 35].

In this work, we focus on a previously unstudied *S. mansoni* glycolytic enzyme, phosphoglycerate mutase (SmPGM). PGM is a key glycolytic enzyme that catalyzes the eighth step of glycolysis: the reversible conversion of 3-phosphoglycerate (3PG) to 2-phosphoglycerate (2PG) [51]. Here, we clone and express active SmPGM and characterize the enzyme’s biochemical properties. By immunolocalization, we confirm that the protein is widely expressed throughout all tissues of the parasites, including in the tegument. Activity assays undertaken using intact, living worms provide evidence that SmPGM is active on the external surface of the intravascular-stage worms and, like SmEno and SmGAPDH, is not secreted or released by cultured worms to any great extent. Finally, we test the hypothesis that external SmPGM may perform some non-glycolytic (moonlighting) functions and we provide evidence that the enzyme has thrombolytic potential for the intravascular worms.

## Materials and methods

### Ethics

All protocols involving animals were approved by the Tufts University Institutional Animal Care and Use Committee (IACUC) under protocols G2021-51 and G2020-112.

### Animals

To obtain *Schistosoma mansoni* parasites, *S. mansoni* cercariae were shed from *Biomphalaria glabrata* snails (strain NMRI, NR-21962, Schistosomiasis Resource Center, Biomedical Research Institute, Rockville, MD, USA) and transformed into schistosomula as previously described [11]. Adult worms were obtained following perfusion of 6- to 8-week-old Swiss-Webster or C57BL/6 mice (*Mus musculus*) 7 weeks post-infection. All parasites were cultured in complete Dulbecco’s modified Eagle’s (DMEM)/F12 medium [supplemented with 10% heat-inactivated fetal bovine serum (FBS), 200 U/mL penicillin, 200 mg/mL streptomycin, 0.2 μM Triiodo-L-thyronine (T_3_), 1 μM serotonin, 8 μg/mL human insulin, and 0.5 mM ascorbic acid] and maintained at 37 °C in an atmosphere of 5% CO_2_ [30].

### PGM activity in live *S. mansoni* parasites

Live *S. mansoni* parasites (~350 schistosomula or one adult male worm per well, in triplicate) were washed and resuspended in 100 μL of standard assay buffer (20 mM HEPES, 135 mM NaCl, 5 mM KCl, 5 mM MgSO_4_, 10 mM glucose). The enzyme reaction was initiated by the addition of substrate (10 mM 3-phosphoglycerate (3PG)), with or without cofactor, as indicated (2,3-bisphosphoglycerate (2,3BPG), 10 mM) and commercially obtained *Saccharomyces cerevisiae* yeast enolase (ScEno, 0.2 μg/reaction, Sigma-Aldrich, Burlington, MA, USA). In this paired reaction, PGM converts 3PG to 2PG and the latter is acted on by enolase to generate phosphoenolpyruvate (PEP) which is measured spectrophotometrically at OD_240_ using a Synergy HT microplate spectrophotometer (BioTek Winooski, VT, USA). Activity measured by parasites incubated in assay alone buffer served as a control.

Schistosomula (~3000) were washed in HBSS and incubated overnight in 600 μL phenol-red-free DMEM (supplemented with 200 U/mL penicillin and 200 μg/mL streptomycin). The next day, the conditioned medium was recovered and tested for PGM activity as described above. Non-conditioned phenol-red-free DMEM was used as a negative control. Worms recovered from conditioned medium were resuspended in fresh phenol-red-free DMEM and any PGM activity displayed by these live parasites was then measured (~500 per well, in triplicate), as described above.

In addition to measuring PGM activity of live schistosomula, as described above, additional schistosomula were homogenized in Hank’s Balanced Salt Solution (HBSS) and PGM activity in this total worm lysate (in triplicate) was measured in standard assay buffer.

### SDS-PAGE and western blot analysis

Parasite homogenates (10 μg protein) were resolved on a 4–20% Mini-PROTEAN^®^ TGX^TM^ sodium dodecyl sulphate-polyacrylamide gel electrophoresis (SDS-PAGE; Bio-Rad, Hercules, CA, USA). Protein was then transferred to an activated semi-dry polyvinylidene difluoride (PVDF) membrane, and the membrane was incubated in blocking buffer (phosphate buffered saline (PBS) + 0.05% Tween-20 (PBST) + 5% dry non-fat milk powder) at room temperature for 1 h. The membrane was incubated with anti-SmPGM or control antisera (generated as described below) diluted 1:10,000 in blocking buffer overnight at room temperature. The membrane was then washed with PBST and incubated with HRP-conjugated goat anti-mouse IgG (H + L) antibody (#170-6516, Bio-Rad) diluted 1:5000 in blocking buffer at room temperature for 1 h. The blots were developed using ECL Western Blotting Detection Reagents (GE Healthcare Bio-Sciences, Piscataway, NJ, USA), following the manufacturer’s instructions. Western blot images were captured using a ChemiDoc Touch Imaging System (Bio-Rad). An equivalent western blot probed with serum from mice immunized with adjuvant alone served as a negative control.

### Suppression of SmPGM gene expression via RNAi

Two SmPGM-specific siRNAs were synthesized (Integrated DNA Technologies Coralville, IA, USA) and used at 6 μM for gene expression knockdown. The sequences for these siRNAs are: SmPGM siRNA #1, 5′-AAGGTATCACTGAGGCTAAACAAGC-3′ and SmPGM siRNA #2: 5′-GAGTGTTTACAATGAAGAAAATCGA-3′. Live schistosomula (~3000) were electroporated with these siRNAs, or an irrelevant control siRNA using methods previously reported [11]. Quantitative reverse transcription PCR (RT-qPCR) was performed 72 h post-electroporation using TaqMan^TM^ Assays (Thermo Fisher Scientific, Waltham, MA, USA²). The following primer sets and reporter probe customized for SmPGM were purchased from Life Technologies (Carlsbad, CA, USA): SmPGM F-primer, 5′-GAAACAGTTTAAGAGCGCTTATCAAGT-3′; SmPGM R-primer, 5′-TCAAGTTCGCATCCAGTTCATAGAC-3′; SmPGM FAM-probe, 5′-ACCCACTGGTATTCCAC-3′. *Schistosoma mansoni* tubulin was used as an endogenous control to compare relative SmPGM expression [43]. Total RNA was extracted using the TRIzol reagent (Invitrogen, Waltham, MA, USA), following the manufacturer’s instructions. cDNA synthesis was performed using 650 ng RNA and a TaqMan^®^ Reverse Transcription kit (Thermo Fisher Scientific), following the manufacturer’s instructions. RT-qPCR reactions were performed using 2 μL of cDNA in a final volume of 20 μL. Reactions were run in triplicate in a StepOne Plus system (Life Technologies). The ΔΔCT method was utilized to determine relative quantification [27].

One week after siRNA treatment, live schistosomula (~1000 per well) were monitored in triplicate for PGM activity, as described earlier. Western blot analysis was conducted as described above. Here, a mouse monoclonal IgG1 anti-recombinant GAPDH antibody (1:5000, MA5-15738, Thermo Fisher Scientific), was used as a protein loading control, as in previous work [35]. Protein signal quantification was performed in ImageJ by measuring the area under the peaks corresponding to each band signal, setting the “No siRNA” band as the reference standard.

### Multiple sequence alignment and phylogenetic tree generation

A multiple sequence alignment (MSA) was generated using sequences of PGMs from the following organisms (accession no.): *Schistosoma mansoni* (UFA46002), *Schistosoma japonicum* (Q8WT66), *Schistosoma bovis* (RTG81826.1), *Homo sapiens* PGAM1 (P18669) and PGAM2 (P15259), *Mus musculus* PGAM1 (Q9DBJ1) and PGAM2 (O70250), *Drosophila melanogaster* (Q9VAN7), *Escherichia coli* 2,3-bisphosphoglycerate-dependent PGM (P62707) and 2,3-bisphosphoglycerate-independent PGM (P37689), *Clonorchis sinensis* (AAX29976.1), *Fasciola hepatica* (THD19385.1), *Caenorhabditis elegans* (G5EFZ1), *Plasmodium falciparum* (Q8IIG6), *Onchocerca volvulus* (I6LDA6), *Biomphalaria glabrata* (XP_013071129.1), *Saccharomyces cerevisiae* (P00950), *Trypanosoma brucei* (Q38AH1), and *Nicotiana tabacum* (P35494). These 19 sequences were imported to BioEdit Sequence Alignment Editor and subjected to ClustalW Multiple alignment to generate a final MSA.

To generate a phylogenetic tree, the MSA file was exported into Molecular Evolutionary Genetics Analysis (MEGA) Version 7.0.21 software and converted into a MEG file. A neighbor-joining phylogenetic reconstruction was created using a 1000-replicate bootstrap test. The Poisson correction method was used to calculate evolutionary distances.

### Cloning and expression of recombinant *S. mansoni* PGM (rSmPGM)

Adult *S. mansoni* cDNA was used as a template to generate a PCR product containing the complete coding DNA for SmPGM (XP_018652140). The following primers were used: SmPGM-F, 5′-TTAACC**GGATCC**AATGGCTCCTTACAGAATTGTGTTTATTCGCC-3′; SmPGM-R, 5′-TCAAAA**CTCGAG**TCATTTCTTTTTTCCCTGGTTCGCCACACG-3′ containing BamHI and XhoI restriction sites, respectively (bold). The purified PCR product was digested with BamHI and XhoI and cloned into a pTrcHisB expression vector (Life Technologies) also digested with BamHI and XhoI. DNA sequencing confirmed the successful generation of the in-frame recombinant plasmid construct (GeneWiz). The PGM construct was subsequently transformed into BL21 Star (DE3) *E. coli* (Thermo Fisher Scientific). Recombinant transformants were cultured in Luria Broth supplemented with ampicillin (100 μg/mL). Recombinant PGM production was induced with 1 mM isopropyl β-D-1-thiogalactopyranoside (IPTG), and rSmPGM were purified by immobilized metal affinity chromatography (IMAC) on Ni-NTA Sepharose columns, following the manufacturer’s instructions (Life Technologies). Eluted protein fractions containing purified rSmPGM was dialyzed against 1X PBS overnight at 4 °C and concentrated via centrifugation in 10 K MWCO Pierce^TM^ Protein Concentrators (Thermo Fisher Scientific). All protein fractions were resolved by 4–20% SDS-PAGE and stained with Coomassie Blue (Bio-Rad). Gel images were captured on a ChemiDoc^TM^ Touch Imaging System (Bio-Rad).

Anion exchange chromatography (AEC) was utilized to further purify rSmPGM eluates from any remaining bacterial contaminants, following the column manufacturer’s instructions (Cytiva, Marlborough, MA, USA). Briefly, IMAC-purified eluted protein fractions containing rSmPGM were dialyzed overnight against Tris buffer (20 mM, pH 7.5) at 4 °C. The dialyzed protein was then applied to an equilibrated 1 mL HiTrap^TM^ Q HP ion exchange column (Cytiva). Next, the column was washed with Tris buffer and bound proteins were then eluted with five column volumes of Tris buffer supplemented with increasing concentrations of NaCl (100 mM–1 M). Eluates and washes were collected during every step for analysis.

Western blotting analysis of purified SmPGM protein extracts and various column fractions was carried out as described above for parasite lysates; however, a commercial anti-PGAM1 antibody (Abcam, ab96622) at 1:5000 dilution was used here as the primary antibody source.

### Generation of rSmPGM antiserum

To generate anti-rSmPGM antiserum, 25 μg of purified recombinant protein (in Freund’s Complete Adjuvant (FCA) #77140, Thermo Scientific) was injected subcutaneously into C57BL/6 mice (*n* = 10). All mice were boosted twice with rSmPGM in Imject^TM^ Freund’s Incomplete Adjuvant (FIA) (#77145, Thermo Scientific) every two weeks. Mice injected with PBS in adjuvant alone, in the same manner, were used to generate a pool of control murine serum. These sera were used for SmPGM immunolocalization, described next.

### Immunolocalization of SmPGM

Live, 7-day-old schistosomula were fixed in 4% paraformaldehyde (PFA) for 5 min at room temperature. The fixed parasites were incubated first with blocking buffer (1% bovine serum albumin (BSA) in PBS) for 1 h and then in serum (diluted 1:50 in blocking buffer) from either rSmPGM-immunized mice or control mice, for 1 h at room temperature. The worms were then washed with PBST before being incubated for 1 h in the dark at room temperature with Alexa Fluor^TM^ 488 goat anti-mouse IgG (H + L) (Invitrogen) diluted in blocking buffer at 1:100. The worms were again washed with PBST and then incubated with 0.3 mM 4’,6-diamidino-2-phenylindole (DAPI) in blocking buffer for 5 min, washed with PBST, mounted in Fluoromount, and placed in clear-bottom 96-well plates. The parasites were viewed using an inverted fluorescent microscope (Eclipse Ti, Nikon and Revolve, Echo).

Frozen sections (6 μm thick) of adult parasites in optimal cutting temperature (OCT) compound (Thermo Fisher Scientific) were hydrated with PBS and incubated with blocking buffer for 1 h at room temperature in a humidified chamber. The sections were processed for immunolocalization using the same techniques described above for whole, fixed schistosomula.

### Characterization of rSmPGM

Functional activity of purified recombinant *S. mansoni* PGM (rSmPGM) was examined using the same paired enzyme reaction (employing yeast enolase (ScEno)) as described earlier in experiments using live parasites. Briefly, the reaction was initiated by adding 100 μL substrate (5 mM 3PG) and with or without cofactor, as indicated (5 mM 2,3BPG) in assay buffer (20 mM Tris-HCL, 0.135 M NaCl, 5 mM KCl, 5 mM MgSO_4_, pH: 7.8) to the enzyme mixture (rSmPGM, 1.0 μg/reaction and ScEno, 0.2 μg/reaction) in assay buffer, in triplicate. PGM activity was monitored in a Synergy HT microplate spectrophotometer (BioTek) at OD_240_ over time. To heat-inactivate the enzyme, rSmPGM was incubated at 100 °C in 1X Dulbecco’s phosphate-buffered saline (DPBS) for 5 min. In some experiments, 2,3BPG concentrations ranged from 0.005 mM to 5 mM, as noted.

To determine the Michaelis-Menten constant (*K*_m_) of rSmPGM for the substrate 3PG, activity was measured at a range of 3PG concentrations (0–5 mM) while cofactor 2,3BPG concentrations remained fixed (5 mM). The *K*_m_ value was calculated using nonlinear regression analysis on GraphPad Prism 5.0 (La Jolla, California).

### Inhibition of rSmPGM activity

rSmPGM activity was measured after 10 min preincubation in the absence or presence of either 1 μM or 10 μM sodium metavanadate (Sigma-Aldrich). PGM activity was then initiated by adding 3PG (5 mM) and enolase (0.2 μg/reaction), and the reaction was monitored as described above, in triplicate.

### Plasminogen binding

Binding of human plasminogen (HYPHEN BioMed) to rSmPGM was measured by ELISA as follows: 1.0 μg/well rSmPGM in bicarbonate buffer (0.05 M, pH: 9.6) was coated onto 96-well Nunc-Immuno microplates (Thermo Fisher Scientific) and incubated at 4 °C overnight. Control wells were coated with 1.0 μg/well BSA (negative control) in carbonate-bicarbonate buffer. All wells were then incubated with blocking buffer (1% BSA in PBST) and incubated at 37 °C for 1 h. Next, wells were incubated with various concentrations (0–3 μg, as indicated) of plasminogen in blocking buffer at 37 °C for 1 h. All wells were then washed with PBST before being incubated with rabbit anti-plasminogen IgG (PA5-34677, Invitrogen) diluted in blocking buffer (1:2000) at 37 °C for 1 h. Next, all wells were washed with PBST and incubated with horseradish peroxidase (HRP)-conjugated donkey anti-rabbit IgG (Cytiva) diluted in blocking buffer (1:5000) at 37 °C for 1 h. After a final washing with PBST, the plates were then developed by adding 100 μL chromogenic substrate 3,3’,5,5’-tetramethylbenzidine (TMB) (Thermo Fisher Scientific) to all wells for 2 min. Color development was stopped by the addition of 100 μL 1 N HCl to all wells. The plates were then read at OD_450_.

### Plasminogen activation

Plasminogen activation assays were performed with rSmPGM, as previously described [35]. Briefly, human plasminogen (3.0 μg), recombinant human tissue plasminogen activator (tPA, 20 ng, HYPHEN BioMed, Neuville-sur-Oise, France) and rSmPGM (0.5–5.0 μg, as indicated) in HBSS were added to 96-well microplates (Corning, NY, USA) to a total volume of 150 μL. BSA (1.0 μg) was used as a negative control. Synthetic plasmin substrate D-Valyl-L-Leucyl-L-Lysine 4-nitroanilide dihydrochloride (4.5 μg, Sigma-Aldrich) in HBSS was then added to all wells. Change in OD_405_ was measured over time using a Synergy HT spectrophotometer that had been pre-heated to 37 °C.

### Statistical analysis

All values are reported as mean ± SEM. Statistical analyses were performed using GraphPad Prism 5.0 (La Jolla, CA, USA). Two-way analysis of variance (ANOVA) with Bonferroni post-tests were used to determine statistical significance for time-course experiments (e.g., activity assays, plasminogen activation assays). One-way ANOVA with Tukey post-tests were used to determine significance for experiments comparing means across multiple groups (e.g., relative gene expression). *P*-values < 0.05 were considered statistically significant.

## Results

### SmPGM is widely expressed in intravascular stage schistosomes including in the tegument

**Figure 1:**
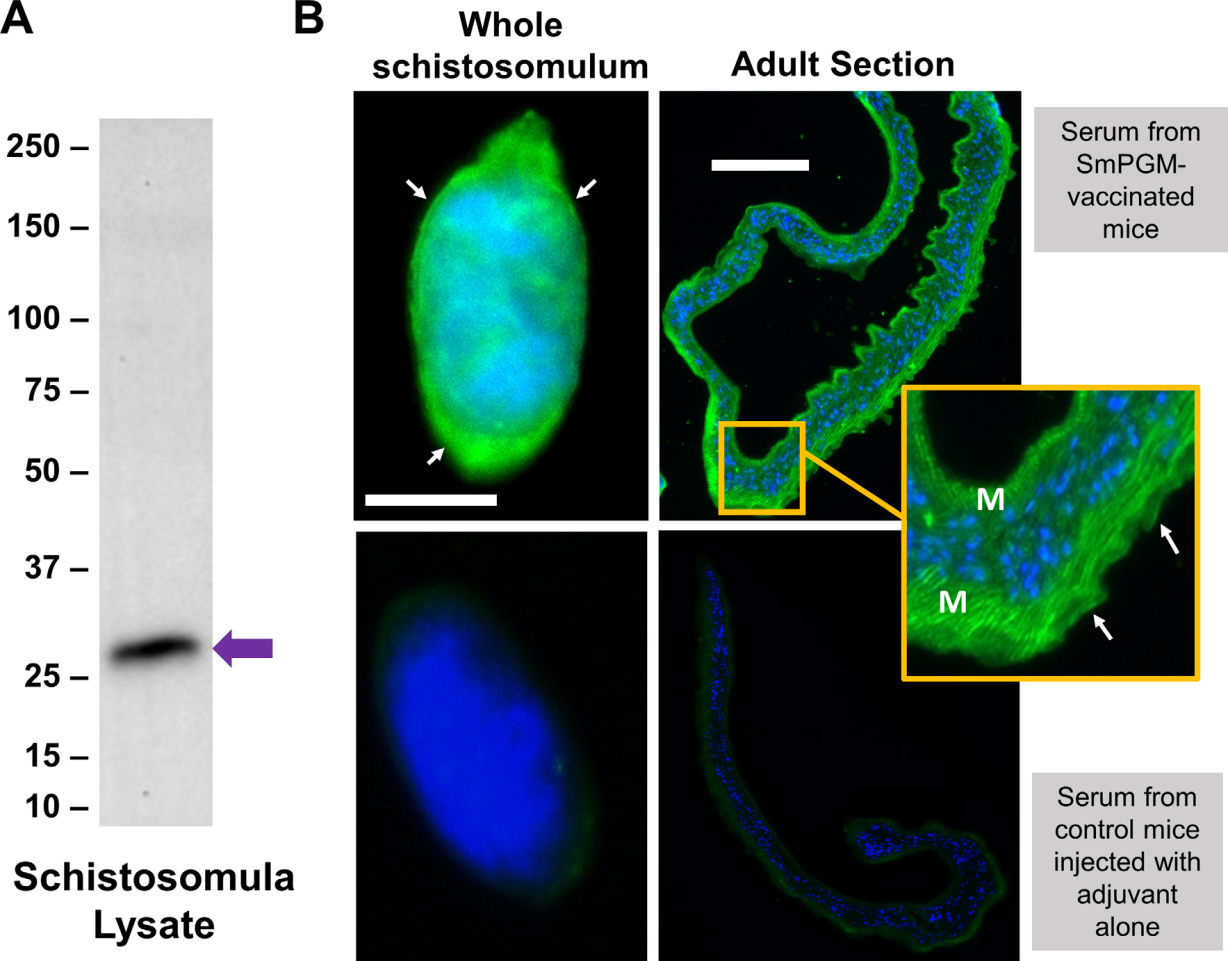
Immunolocalization of SmPGM in *Schistosoma mansoni* schistosomula and adult worms. (A) Western blot analysis demonstrating binding of antibodies from rSmPGM-immunized mice to a protein of the expected size of native SmPGM in a *Schistosoma mansoni* schistosomula lysate (arrow). Blots probed with sera derived from mice immunized with adjuvant alone showed no binding to parasite lysate (not shown). Numbers to the left indicate markers representing molecular mass in kDa. (B) Indirect immunofluorescent labeling of native SmPGM (green) in a whole fixed *S. mansoni* schistosomulum (top left) and a section of an adult *S. mansoni* worm (top right) using sera from mice immunized with rSmPGM. Tegument staining is indicated by white arrows. Sub-tegumental muscle staining is indicated (M) in the inset yellow-bordered box. Counterstaining with DAPI highlights nuclei (blue). Lower images show a schistosomulum (left) and an adult section (right) incubated with serum from mice injected with adjuvant alone as negative controls. Scale bars: 100 μm.

When anti-SmPGM antiserum, generated by immunizing mice with recombinant SmPGM, is used to probe a western blot containing schistosomula lysate, a prominent band of the expected size of SmPGM (~28 kDa) is detected (Fig. 1A, arrow). Western blots probed with sera derived from control mice injected with adjuvant alone showed no binding to parasite lysate (not shown). To localize SmPGM within the parasites, whole, fixed schistosomula that had been cultured for >7 days (Fig. 1B, upper left) and sections of adult parasites (Fig. 1B, upper right) were stained with the anti-SmPGM antiserum. Robust staining is seen throughout the parasites, especially in the sub-tegumental muscle layer in adult sections (inset, M), as well as in the tegument of adults and schistosomula (white arrows). Control parasites incubated with sera from mice injected with Freund’s adjuvant alone showed low background staining (Fig. 1B, lower panels).

### Live schistosome parasites exhibit functional PGM activity on their surface

**Figure 2:**
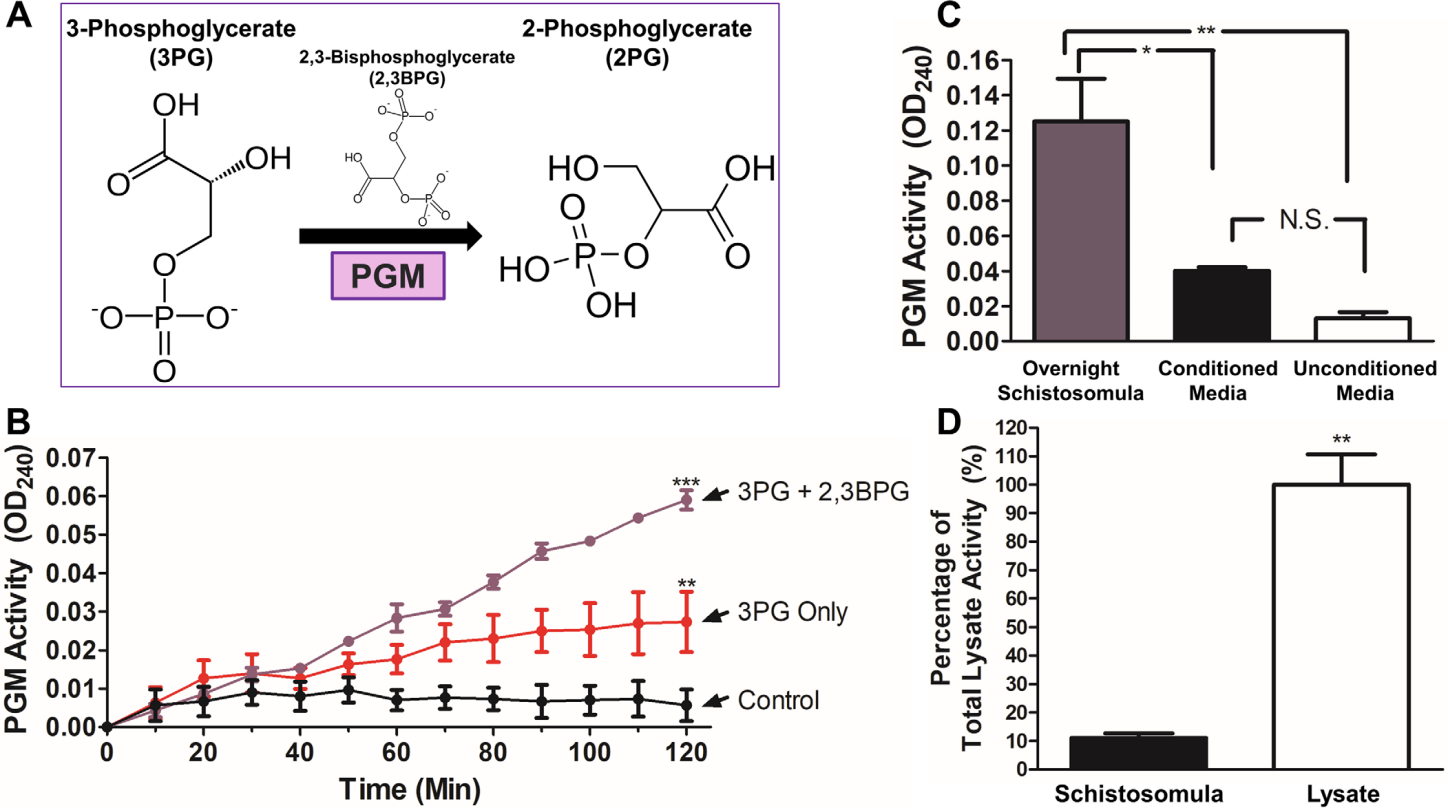
Characterization of native SmPGM. PGM catalyzes the eighth step of glycolysis illustrated in (A) 3-phosphoglycerate (3PG) is converted to 2-phosphoglycerate (2PG). 2,3-bisphosphoglycerate (2,3BPG, depicted above the arrow) is required as a cofactor in some PGMs (i.e., cofactor-dependent PGMs, or dPGMs). (B) PGM activity over time displayed by live schistosomula when incubated with 3PG and 2,3BPG (purple line) or 3PG only (red line). Activity measured in parasites incubated in assay buffer alone served as a control (black line). (C) PGM activity of live schistosomula (purple bar) following overnight incubation in medium compared with PGM activity in conditioned medium (black bar) and unconditioned medium (white bar). (D) PGM activity of live schistosomula (black bar) compared to PGM activity by an equivalent schistosomula lysate (white bar) set at 100%. Significant differences are denoted by ***p < 0.001, **p < 0.01, *p < 0.05.

PGM converts 3-phosphoglycerate (3PG) to 2-phosphoglycerate (2PG) [51] in a reaction depicted in Figure 2A. Also depicted above the arrow in Figure 2A is cofactor 2,3-bisphosphoglycerate (2,3BPG). As noted in Materials and methods, 2PG is converted by enolase to phosphoenolpyruvate (PEP). In our analysis of PGM function in this work, it is PEP generation that is monitored here at OD_240_ over time. Using this PGM-enolase paired assay, it is clear that living, intact *S. mansoni* schistosomula do display PGM activity. As shown in Figure 2B, live worms, incubated with substrate 3PG, cofactor 2,3BPG, and commercially obtained yeast enolase (ScEno) show a sustained increase in OD_240_ over time (purple line, 3PG + 2,3BPG) compared to parasites incubated in the absence of substrate 3PG (black line, Control). Note that live schistosomula still exhibited significant surface PGM activity in the presence of 3PG but *without* 2,3BPG (Fig. 2B, red line, 3PG Only). It is clear that adding 2,3BPG significantly boosts SmPGM activity (*p* < 0.001, 120 min timepoint). Activity measured either in the presence of 3PG alone or in the presence of 3PG plus 2,3BPG both differ significantly from the control condition (*p* < 0.001, 120 min timepoint).

PGM activity was measured in a conditioned, phenol-red-free culture medium that contained parasites (500 schistosomula) incubated overnight. Figure 2C shows that this conditioned medium (black bar) displays very low PGM activity, whereas live schistosomula recovered from this medium display robust activity (purple bar, *p* < 0.05). Activity detected in conditioned medium does not differ significantly from that displayed by fresh (unconditioned) medium (white bar). We additionally tested if SmPGM was secreted from worms that were incubated in a harsher solution (enzyme assay buffer) for 1 h and we found essentially no activity in this solution but, again, robust activity was displayed by the worms that were recovered from the buffer after the 1 h incubation (Supplementary Fig. 1).

The PGM activity of live schistosomula was compared with the PGM activity from a lysate of equivalent numbers of whole parasites (set at 100%). As shown in Figure 2D, lysate exhibits significantly greater PGM activity (white bar, *p* < 0.01) compared to that displayed by live worms (black bar); mean live worm surface PGM activity represents 11 ± 2% of total parasite PGM activity.

### Suppression of SmPGM gene expression

**Figure 3:**
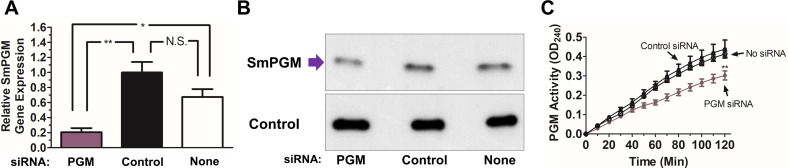
SmPGM gene suppression using RNA interference. (A) Relative SmPGM gene expression in schistosomula 72 h after electroporation with siRNA targeting SmPGM (purple bar, PGM), control siRNA (black bar, Control) or no siRNA (white bar, None) as assessed by RT-qPCR. Significant differences are denoted by *p < 0.05, **p < 0.01 (one-way ANOVA with Tukey’s post-test). (B) Western blot analysis of SmPGM protein (purple arrow) in schistosomula lysates 7 days after electroporation with siRNA targeting SmPGM (left), control siRNA (middle), or no siRNA (right) as indicated. Blots were probed with anti-GAPDH monoclonal antibody (lower panel, Control) to show that all lanes received roughly equivalent amounts of protein. (C) Mean SmPGM activity in live schistosomula tested 7 days after electroporation with siRNA targeting SmPGM (purple line), control siRNA (black line, filled circles), or no siRNA (black line, empty circles). Significant differences for all time points after 70 min are denoted by **p < 0.01 (two-way ANOVA with Bonferroni post-test).

SmPGM gene expression was suppressed *ex vivo* using RNA interference (RNAi). As shown in Figure 3A, treatment of live schistosomula with siRNAs targeting SmPGM resulted in significant (~80%) gene suppression (purple bar) as measured by real-time quantitative polymerase chain reaction (RT-qPCR) analysis compared to parasites treated with a control siRNA (black bar, *p* < 0.01) or parasites treated with no siRNA (white bar, *p* < 0.05).

Western blot analysis (Fig. 3B) reveals that notably less SmPGM protein (~38% reduction) is detected in lysates of parasites treated with the SmPGM siRNA (purple arrow) compared to parasites treated with a control siRNA (Control) or no siRNA (None), as indicated. Identical lysates were probed with a control antibody (that detects the glycolytic enzyme GAPDH – Fig. 3B, bottom) to demonstrate that each extract contained roughly equivalent amounts of protein.

Finally, Figure 3C shows that live parasites treated with siRNA targeting SmPGM show significantly reduced surface PGM activity (purple line) compared to parasites treated with either control (*p* < 0.001 at 120 min) or no siRNA (*p* < 0.01 at 120 min). Parasites treated with siRNA targeting SmPGM showed no noticeable differences in motility, morphology, or viability during culture for 7 days.

### Analysis of SmPGM

Examination of the published *S. mansoni* transcriptome revealed the presence of a phosphoglycerate mutase homolog whose GenBank accession number is XM_018797061 [38]. Using this sequence as a guide, we designed primers to amplify the complete SmPGM coding sequence which was then purified and sequenced at GeneWiz Inc. No differences in sequence were detected between XM_018797061 and our PCR product (complete SmPGM coding sequence, GenBank accession number OK490368.1).

**Figure 4:**
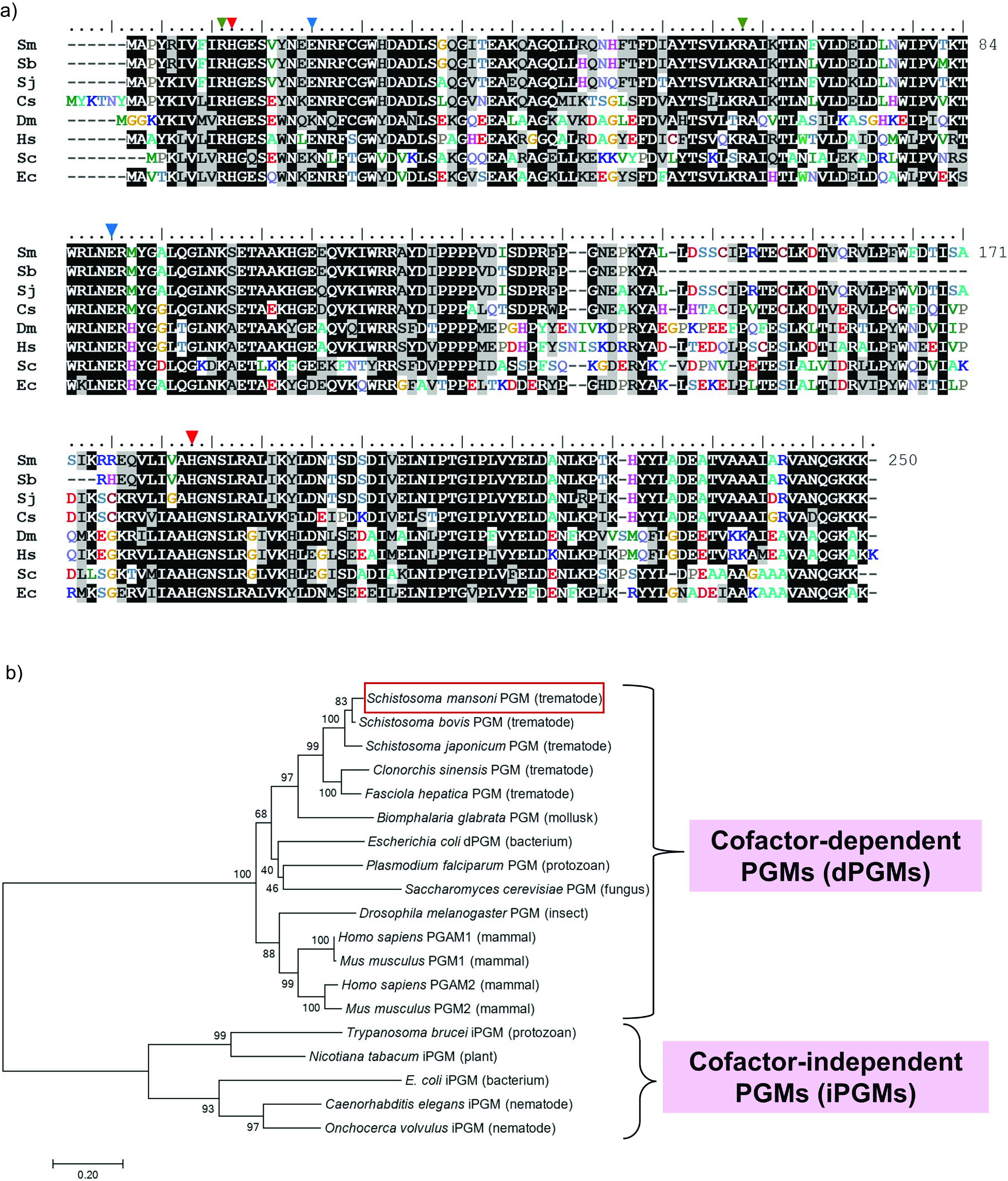
(A) Sequence alignment of *Schistosoma mansoni* PGM (Sm) and select dPGMs from other organisms. Identical amino acid residues between sequences are shaded in black. Similar amino acid residues between sequences are shaded in gray. Important conserved residues are marked with arrowheads: the histidine residues (red), arginine residues (green), and glutamate residues (blue) have been reported to stabilize the active site and participate in binding and catalysis of the substrate 3-phosphoglycerate. The accession numbers of the aligned PGMs are as follows: *Schistosoma mansoni* Sm (OK490368.1), *Schistosoma bovis* Sb (RTG81826.1), *Schistosoma japonicum* Sj (Q8WT66), *Clonorchis sinensis* Cs (AAX29976.1), *Drosophila melanogaster* Dm (Q9VAN7), *Homo sapiens* Hs PGAM1 (P18669), *Saccharomyces cerevisiae* Sc (P00950), *Escherichia coli* Ec 2,3-bisphosphoglycerate-dependent PGM (P62707). (B) Phylogenetic tree of phosphoglycerate mutases. A phylogenetic tree of phosphoglycerate mutases constructed by Molecular Evolutionary Genetics Analysis (MEGA) Version 7.0.21 software using amino acid sequences obtained from the UniProt database. The optimal tree with the sum of branch length = 5.29 is shown. The scale bar represents the number of substitutions between sequences. Evolutionary distances were computed using the Poisson correction method. The analysis involved 19 amino acid sequences. The species names and UniProt accession numbers for the sequences are as follows: *Schistosoma mansoni* (UFA46002), *Schistosoma japonicum* (Q8WT66), *Schistosoma bovis* (RTG81826.1), *Homo sapiens* PGAM1 (P18669) and PGAM2 (P15259), *Mus musculus* PGAM1 (Q9DBJ1) and PGAM2 (O70250), *Drosophila melanogaster* (Q9VAN7), *Escherichia coli* 2,3-bisphosphoglycerate-dependent PGM (P62707) and 2,3-bisphosphoglycerate-independent PGM (P37689), *Clonorchis sinensis* (AAX29976.1), *Fasciola hepatica* (THD19385.1), *Caenorhabditis elegans* (G5EFZ1), *Plasmodium falciparum* (Q8IIG6), *Onchocerca volvulus* (I6LDA6), *Biomphalaria glabrata* (XP_013071129.1), *Saccharomyces cerevisiae* (P00950), *Trypanosoma brucei* (Q38AH1), and *Nicotiana tabacum* (P35494).

SmPGM is predicted to have 250 amino acids with a predicted molecular mass of 28,426 Da and isoelectric point (pI) of 7.71. Figure 4A shows an alignment of PGM sequences from a variety of phylogenetically different organisms. Identical residues are shaded in black, while similar residues are shaded in gray. SmPGM shares ~58% sequence identity with human PGM. Several key conserved residues are marked with colored arrowheads. Red arrowheads indicate catalytic histidine residues (His11, His184) that transfer a phosphoryl group to the second carbon (C2) of 3PG bound within the active site to form diphosphorylated 2,3BPG. This then donates the phosphoryl group on its third carbon (C3) to generate 2PG and regenerates the active site [51]. Green arrowheads mark arginine residues (R10, R62) that are predicted to help maintain optimal enzyme activity. Blue arrowheads mark glutamate residues (E19, E89) whose carboxyl moieties act as proton-withdrawing groups flanking 3PG within the active site [51].

BLAST analysis of the amino acid sequence predicts SmPGM to be a 2,3BPG-dependent PGM and places the enzyme in the histidine acid phosphatase (HAP) superfamily – a large group of enzymes that share a conserved active site featuring a histidine that is phosphorylated during the enzymatic reaction [41]. MSA analysis of SmPGM with PGMs from a variety of other organisms yielded the phylogenetic tree depicted in Figure 4B. SmPGM (red box, top) clearly associates closely with predicted homologs from other schistosomes and other trematodes. SmPGM also clearly falls within the cofactor (2,3-bisphosphoglycerate)-dependent PGM (dPGM) group as distinct from the cofactor-independent PGMs (iPGMs).

### Cloning, expression, and purification of recombinant SmPGM (rSmPGM)

**Figure 5:**
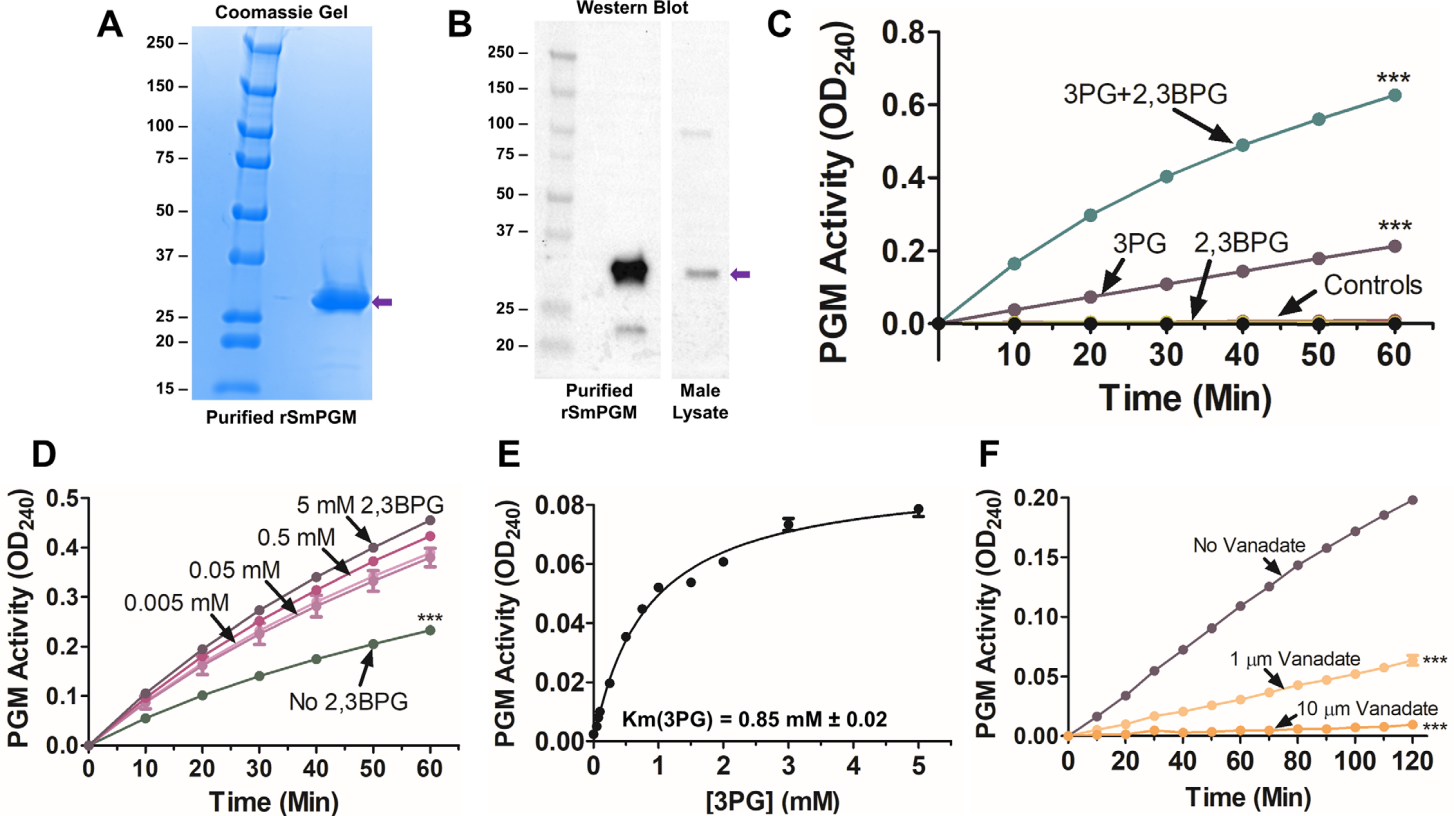
Expression, purification, and characterization of rSmPGM. (A) Coomassie Blue-stained gel showing purified rSmPGM running at ~28 kDa (purple arrow). Molecular mass markers are shown (left); numbers represent kDa. (B) Western blot showing pure rSmPGM (left) detection using a commercial anti-PGM antibody. A prominent protein band running at the expected size of SmPGM is detected in adult *S. mansoni* male lysate (right, purple arrow). Numbers to the left of the Coomassie and western blots indicate molecular markers representing molecular mass in kDa. (C) rSmPGM activity in the presence of either substrate 3PG only (purple line) or 3PG plus cofactor 2,3BPG (teal line, top). One notable control includes reaction in the presence of 2,3BPG only. Other controls include samples lacking 3PG, enolase, or rSmPGM. ***p < 0.001 (two-way ANOVA with Bonferroni post-test). (D) rSmPGM activity in the absence of 2,3BPG (3PG Only, green line) or supplemented with 0.005–5.0 mM 2,3BPG (pink and purple lines). ***p < 0.001 (two-way ANOVA with Bonferroni post-test). (E) Michaelis-Menten kinetic curve shown for the substrate 3PG. The *K*_m_ value shown is the mean from three independent experiments. (F) Inhibition of rSmPGM activity by 1 μM (light orange line) or 10 μM (dark orange line) sodium metavanadate. Samples with no inhibitor (purple line) served as a control. ***p < 0.001 (two-way ANOVA with Bonferroni post-test at 120 min).

rSmPGM, with an in-frame N-terminal poly-His motif, was expressed in BL21 Star (DE3) *Escherichia coli* and was purified first by standard immobilized metal affinity chromatography (IMAC) and then via anion exchange chromatography (AEC). As shown in Figure 5A, rSmPGM resolves by SDS-PAGE as a single, dominant band at ~28 kDa. As shown in Figure 5B, commercial anti-human PGM antibody (anti-PGAM1, Abcam) binds to both purified rSmPGM and to a protein of about the same molecular mass, likely native SmPGM, in an adult male *S. mansoni* lysate (purple arrow).

### Characterization of rSmPGM

Purified rSmPGM was tested for classical phosphoglycerate mutase activity in the standard paired enzyme reaction described in Materials and methods. As shown in Figure 5C, rSmPGM is active and displays the same characteristics as seen in whole worms. In other words, rSmPGM is active in the absence of cofactor 2,3BPG (purple line, 3PG) but this activity increases significantly upon the addition of cofactor (upper (teal) line, 3PG + 2,3BPG, *p* < 0.001). Notably, there is no activity detected when rSmPGM is incubated with cofactor 2,3BPG but without 3PG (yellow line, 2,3BPG). Other control incubations that yield no activity include a mixture of all reagents except SmPGM, a mixture of all reagents except yeast enolase, and a mixture of all reagents with no added enzymes (black line, all such Controls).

Figure 5D shows that 2,3BPG significantly enhances rSmPGM activity at a wide range of concentrations, and to roughly the same degree (pictured are 0.005 mM–5.0 mM 2,3BPG, pink and purple lines), compared to the activity seen in the absence of 2,3BPG (green line, No 2,3BPG, *p* < 0.001). Figure 5E shows that the Michaelis-Menten constant (*K*_m_) of rSmPGM for 3PG is ~0.85 ± 0.02 mM. Figure 5F shows that vanadate significantly inhibits rSmPGM activity in a dose-dependent manner (*p* < 0.001). Note that vanadate can impede yeast enolase (ScEno) activity but only at concentrations much greater than used here (>1 mM, data not shown).

### rSmPGM binds plasminogen and enhances plasmin generation

**Figure 6:**
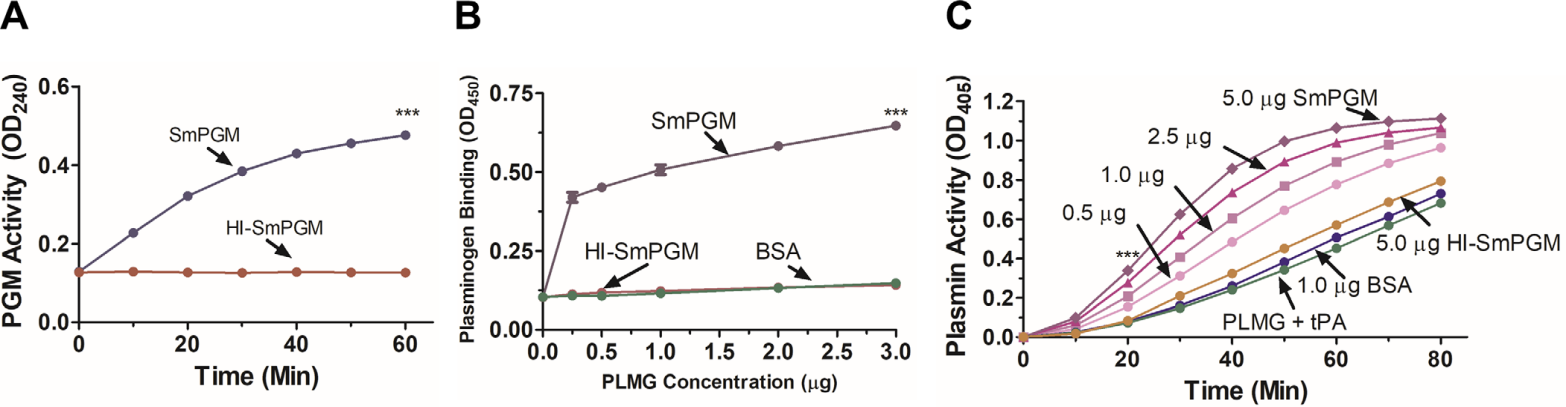
rSmPGM binds to plasminogen and promotes plasmin generation. (A) SmPGM activity of heat-inactivated SmPGM (HI-SmPGM, red line) compared to non-heat-treated SmPGM (blue line) ***p < 0.001 (two-way ANOVA with Bonferroni post-test). (B) Plasminogen (PLMG, at increasing concentration) binding to purified rSmPGM (0.5 μg, purple line) versus heat-inactivated rSmPGM (HI-SmPGM, 5.0 μg, red line) or control protein (BSA, 0.5 μg, green line) detected by ELISA. Significant differences between PLMG binding to rSmPGM versus heat-inactivated (HI) rSmPGM or BSA at all concentrations of PLMG are denoted by ***p < 0.001 (two-way ANOVA with Bonferroni post-test). (C) Plasmin activity detected in the presence of tPA and PLMG alone (green line) or supplemented with increasing amounts of purified SmPGM (0.5–5.0 μg, as indicated) or BSA (1.0 μg, blue line) or HI-SmPGM (5.0 μg, orange line) over time. Significant differences at all time points beyond 20 min compared to controls are denoted by ***p < 0.001 (two-way ANOVA with Bonferroni post-test).

Figure 6A shows that heat-inactivated (HI)-rSmPGM is not enzymatically active compared to the non-heat-treated enzyme (*p* < 0.001 at 120 min). Figure 6B shows that, as measured by ELISA, rSmPGM binds to plasminogen (PLMG). In contrast, HI-rSmPGM does not bind to PLMG, nor does control protein BSA (*p* < 0.001 for all PLMG concentrations, SmPGM *vs.* HI-SmPGM/BSA). Plasmin is generated by the action of tPA on plasminogen (PLMG) (Fig. 6C, green line, PLMG + tPA). Plasmin generation is significantly enhanced by the addition of increasing concentrations of rSmPGM (0.5–5.0 μg, pink and purple lines) in a dose-dependent manner. At all rSmPGM concentrations, plasmin generation is significantly enhanced after the 10 min timepoint (*p* < 0.001) in the presence of the recombinant enzyme. Control protein (1.0 μg BSA, blue line) has essentially no impact on plasmin formation in the presence of tPA. Heat-inactivation of rSmPGM (5.0 μg) causes it to lose its ability to activate plasmin (orange line) compared to the non-heat-treated enzyme.

## Discussion

In this study, we focus on a previously uncharacterized *S. mansoni* glycolytic enzyme – phosphoglycerate mutase (SmPGM). Of special interest is our finding that this enzyme, in addition to being found in the cytosol of worm cells (as expected for a classical glycolytic enzyme), is also found at the worm surface. Immunofluorescent labeling of fixed parasites using an anti-SmPGM antiserum revealed strongest staining in the peripheral muscle layer of adult worms. In addition, SmPGM localized to the entire body of the worm, including in the tegument, which is as expected given that glycolysis is a pathway common to all tissues. Indeed, a single cell RNA-seq atlas of *S. mansoni* revealed SmPGM to be in high abundance in muscle and neuronal cell populations [49]. At this level of analysis, our localization does not tell us if the protein is exposed at the surface of the worms. However, a review of the literature reveals that PGM is identified in 9 out of 11 independent schistosome tegumental membrane proteomic studies, lending support to the notion that the protein can be host-interactive [36].

An important question examined here is whether SmPGM is secreted by parasites, or if it can remain associated with the exterior of the worms (or both). Since enzymatic shaving of live adult worms released SmPGM [7], this suggests that at least some SmPGM is associated with the external surface of the parasites [7]. However, SmPGM (and homologs in other schistosomes) have also been detected in some – but not all – proteomic analyses of schistosome egg, cercarial, schistosomula, and adult worm secretions [5, 25, 42]. We set out to test the hypothesis that (at least some) SmPGM is firmly associated with the surface of the worms and is not released from the worms due to leakage of cytosol following tegument damage that could occur during handling or culture [50]. To do this, we first tested for SmPGM secretion by comparing the PGM enzyme activity displayed by intact, live worms with that of media in which parasites had been incubated overnight. While minimal activity was detected in the conditioned medium, the activity displayed by live worms that were recovered from that medium was markedly and significantly higher. We additionally tested if SmPGM was secreted from worms that were incubated in an enzyme assay buffer for 1 h. Here again, we found essentially no activity in this solution but robust activity was displayed by the worms that were recovered from the buffer after the 1 h incubation. From these data, we conclude that SmPGM is associated with the worms’ outer tegumental membranes and does not “leak” in any appreciable manner under the conditions used here. Finally, we found that surface SmPGM activity accounted for just a small proportion (~11%) of total PGM activity detected in total worm lysates, and this is expected since glycolysis is a conserved, widely-expressed, cytosolic metabolic pathway [8].

We successfully suppressed SmPGM gene expression using RNAi: schistosomula electroporated with siRNAs targeting SmPGM showed significantly lowered levels of PGM mRNA compared to those administered control siRNA or no siRNA. Levels of rSmPGM protein were also decreased in parasites electroporated with siRNA targeting SmPGM. These worms also displayed significantly reduced surface PGM activity compared to controls. These data show that the SmPGM gene encodes the protein whose activity we are measuring on the worm surface.

PGM knockdown in human pancreatic cancer cells enhances apoptosis, and inhibits cellular proliferation, migration, and invasion [48]. In contrast, SmPGM-suppressed schistosomula did not show noticeable changes in viability or morphology over the course of this experiment. This mirrors similar phenomena observed in live *S. mansoni* parasites whose GAPDH and enolase genes were knocked down, indicating, surprisingly, that worms do not require robust glycolytic activity to survive in culture [13, 35].

As PGMs are classified as either dependent or independent on the presence of the cofactor 2,3BPG for activity [14], we set out to characterize SmPGM phylogenetically and biochemically in the context of other known PGMs. Nematodes (e.g., the non-parasitic worm *C. elegans* and the parasites *Onchocerca volvulus*, *Brugia malayi*, and *Wuchereria bancrofti*) have all been reported to possess only cofactor-independent PGMs (iPGMs) [12, 40]. In contrast, trematodes and cestodes have cofactor-dependent PGMs (dPGMs) similar to those of their mammalian hosts [20, 45]. The predicted size of SmPGM immediately suggests that it is a dPGM, and SmPGM amino acid sequence analysis confirms this. SmPGM possesses conserved residues common amongst dPGMs that are considered important for optimal active site function, including conserved histidine, arginine, and glutamate residues that surround 3PG in the active site and facilitate its conversion to 2PG [51]. Phylogenetic analysis clearly categorizes SmPGM with other dPGMs, including that of the previously characterized dPGM of the related trematode parasite *C. sinensis* (CsPGM) [44].

Purified recombinant SmPGM (rSmPGM) resolves by SDS-PAGE as a dominant band at ~28 kDa, about the molecular mass of described dPGMs from other organisms [15, 21, 44]*.* rSmPGM was tested for enzyme activity in the presence and absence of added 2,3BPG; surprisingly, rSmPGM exhibited activity in the absence of 2,3BPG despite its classification as a dPGM. However, mirroring the activity displayed by live parasites, rSmPGM activity was significantly enhanced in the presence of 2,3BPG. Adding increasing amounts of 2,3BPG did not notable enhance enzyme activity, suggesting that a small amount of cofactor is sufficient for optimal SmPGM activity. SmPGM is not unique in this regard: rabbit PGM was similarly found to display some activity in the absence of 2,3BPG [45]. Purified recombinant human PGAM1, which also classifies as a dPGM, was reported to show comparable levels of enzyme activity in the presence and absence of 2,3BPG [20]. While 2,3BPG is not required for SmPGM activity, its ability to enhance SmPGM activity may facilitate more efficient glycolysis. Since SmPGM was unable to convert 2,3BPG into 3PG, this enzyme notably lacks the phosphatase activity that some other dPGMs have been reported to possess [14]. We do not know if SmPGM exhibits synthase activity (i.e., conversion of 1,3BPG to 2,3BPG), as do some other dPGMs [33]. The *K*_m_ value of SmPGM for 3PG (0.85 mM ± 0.2) was of a similar magnitude to that reported for e.g., *C. sinensis* PGM (CsPGM, 0.98 mM) [44] and *E.coli* dPGM (0.2 mM) [15]. rSmPGM activity was significantly blocked in a dose-dependent manner by vanadate, a known and potent inhibitor of dPGMs [4]. Vanadate also inhibited CsPGM at similar concentrations to those used here [4]. Since vanadium compounds can be used as anti-parasitics (against e.g., protozoan parasites such as *Trypanosoma cruzi*, *Leishmania* spp., *Entamoeba histolytica*) [34], this raises the possibility that vanadate could be developed as a therapeutic against schistosomiasis (bearing in mind that host vanadate-sensitive dPGMs must remain functional).

In addition to their ancient and well-described catalytic function in glycolysis, some PGMs have been known to exhibit secondary, or “moonlighting” functions. In plant cells, PGM has been found in a substrate-channeling complex (also containing the glycolytic enzymes enolase and pyruvate kinase) involved in the colocalization of mitochondria and chloroplasts within cells [53]. PGMs have also been detected on the external surfaces of other organisms. For instance, PGMs isolated from *Streptococcus suis* cell wall/secreted protein extracts was identified as a novel binding protein of the extracellular matrix (ECM) proteins fibronectin and collagen type I [52]. The surface-displayed PGM of *Mycoplasma pneumoniae* was reported to bind to the ECM proteins laminin, vitronectin, and fibrinogen [16]. The pathogenic fungus *Candida albicans* has been shown to use surface-localized PGM (CaPGM) to facilitate adhesion to human endothelial cells and keratinocytes by binding to the surface ligand vitronectin, a component of the ECM and a complement regulator [28]. CaPGM has been reported to also bind high molecular weight kininogen (HK), which could enhance virulence *in vivo* by triggering the proinflammatory kinin-generation system at the cell surface and facilitating fungal invasion at the site of infection [22]. CaPGM was also identified as one of several surface-bound proteins that bind to granular enzymes (e.g., elastase, myeloperoxidase, lactotransferrin) derived from neutrophil extracellular traps (NETs) and enhances human epithelial cell destruction [23].

PGM has been reported to bind to plasminogen (PLMG), the zymogen precursor to the fibrinolytic enzyme plasmin, in several organisms including commensals (oral *Streptococcus* spp. [24], *Bifidobacterium lactis* [3]) and pathogens (*M. pneumoniae* [17], *C. albicans* [10, 37]). Importantly, the PGM of *Schistosoma bovis* is a known PLMG binder [39]*.* Some of these organisms (*B. lactis*, *M. pneumoniae*, and *C. albicans*) have been reported to bind PLMG directly to their cell surfaces, possibly via external PGM [3, 10, 17]. Plasmin is thought to be activated by some pathogens to facilitate invasion (by e.g., plasmin-mediated destruction of ECM proteins), to modulate hemostasis, and to evade the immune response (via e.g., disruption of complement function) [1, 2, 6]. Additionally, *M. pneumoniae* PGM was shown to convert PLMG to plasmin in the presence of PLMG activators, which led to *in vitro* degradation of human ECM proteins fibrinogen [17] and vitronectin [16]. *Candida albicans* PGM can bind to the complement regulators factor H and factor H-like protein 1 [19], factors that retain biological regulatory activity while bound to PGM, leading to the conclusion that *C. albicans* uses surface-localized PGM to recruit complement regulators, thus mediating immune evasion [37]. There is growing evidence that schistosome tegumental glycolytic enzymes engage in moonlighting functions as well [36]. Our laboratory has shown that recombinant *S. mansoni* enolase (SmEno) and GAPDH (SmGAPDH) both bind to and promote the activation of plasminogen in the presence of tPA [13, 35]. We show here that rSmPGM similarly binds to and activates PLMG in a dose-dependent manner, in the presence of tPA *in vitro.* Heat-inactivation of rSmPGM ablated not just enzyme activity but also PLMG binding and activation, suggesting that the native 3-dimensional conformational state of the protein is important for its functional interaction with PLMG. Like SmEno and SmGAPDH, surface-exposed SmPGM may also help prevent blood clot formation around schistosomes within the vasculature of their hosts by binding host PLMG and promoting its activation to plasmin, which efficiently cleaves fibrin blood clots [46]. Conceivably, schistosomes could also use tegumental PGM in a manner similar to *C. albicans*, i.e., by binding complement regulators like factor H and factor H-like protein 1 to prevent complement attack at the worm surface [37]. These moonlighting functions could promote parasite survival *in vivo* by helping the worms avoid potentially damaging host hemostatic and immune responses.

In summary, we have shown that SmPGM is active on the external surface of *S. mansoni* parasites and that RNAi suppression of SmPGM reduces gene expression and enzyme activity in live worms. We have phylogenetically categorized SmPGM as a cofactor-dependent PGM (dPGM) and have demonstrated that the activity of the ~28 kDa rSmPGM protein is enhanced in the presence of 2,3BPG and is inhibited by vanadate. We have also shown that rSmPGM binds to the zymogen PLMG and promotes its activation to the fibrinolytic enzyme plasmin in the presence of tPA *in vitro*. PGM’s multifaceted roles as both a glycolytic enzyme and PLMG binder make it a promising therapeutic target; future work on developing SmPGM-specific inhibitory drugs and SmPGM vaccines may advance solutions to treat and eliminate schistosomiasis.

## Supplementary materials

The Supplementary materials of this article are available at https://www.parasite-journal.org/10.1051/parasite/2022042/olm.**Figure S1:**
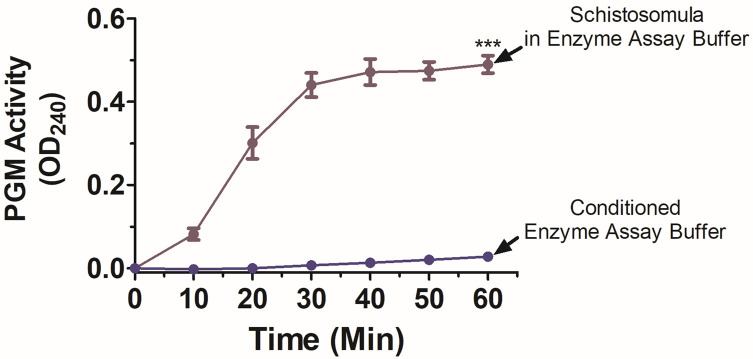
PGM activity of live schistosomula that had been incubated in enzyme assay buffer (purple line) for 1 h compared with PGM activity in that assay buffer (dark blue line). Significantly greater activity is displayed by worms, while essentially no activity is detected in the buffer. ***p < 0.001 (60 min timepoint, two-way ANOVA with Bonferroni post-test).
